# Natural Co-Occurrence of Mycotoxins in Foods and Feeds and Their *in vitro* Combined Toxicological Effects

**DOI:** 10.3390/toxins8040094

**Published:** 2016-03-26

**Authors:** Marie-Caroline Smith, Stéphanie Madec, Emmanuel Coton, Nolwenn Hymery

**Affiliations:** Université de Brest, EA 3882 Laboratoire Universitaire de Biodiversité et d’Ecologie Microbienne, ESIAB, Technopôle Brest-Iroise, 29280 Plouzané, France; marie-caroline.smith@univ-brest.fr (M.-C.S.); stephanie.madec@univ-brest.fr (S.M.); emmanuel.coton@univ-brest.fr (E.C.)

**Keywords:** mycotoxins, foodstuffs, regulations, co-occurrence, combined toxicological effects

## Abstract

Some foods and feeds are often contaminated by numerous mycotoxins, but most studies have focused on the occurrence and toxicology of a single mycotoxin. Regulations throughout the world do not consider the combined effects of mycotoxins. However, several surveys have reported the natural co-occurrence of mycotoxins from all over the world. Most of the published data has concerned the major mycotoxins aflatoxins (AFs), ochratoxin A (OTA), zearalenone (ZEA), fumonisins (FUM) and trichothecenes (TCTs), especially deoxynivalenol (DON). Concerning cereals and derived cereal product samples, among the 127 mycotoxin combinations described in the literature, AFs+FUM, DON+ZEA, AFs+OTA, and FUM+ZEA are the most observed. However, only a few studies specified the number of co-occurring mycotoxins with the percentage of the co-contaminated samples, as well as the main combinations found. Studies of mycotoxin combination toxicity showed antagonist, additive or synergic effects depending on the tested species, cell model or mixture, and were not necessarily time- or dose-dependent. This review summarizes the findings on mycotoxins and their co-occurrence in various foods and feeds from all over the world as well as *in vitro* experimental data on their combined toxicity.

## 1. Introduction

Mycotoxins are secondary fungal metabolites mainly produced by species from the *Aspergillus*, *Penicillium*, and *Fusarium* genera. These toxins are found all around the world as natural contaminants in numerous commodities of plant origin, especially in cereals grains, but also in nuts, oilseeds, fruits, dried fruits, vegetables, cocoa and coffee beans, wine, beer, as well as herbs and spices. Mycotoxins can also be found in animal-derived food if animals eat contaminated feed, namely meat, eggs, milk, and milk derivatives [1,2].

Mycotoxin production, especially on grains, is highly dependent on pre and/or postharvest environmental factors (e.g., temperature and moisture content). Climate represents the key factor in mycotoxin and fungal occurrence. Mycotoxins are climate-dependent compounds but several factors can affect their presence, such as bioavailability of micronutrients, insect damage making it a complex and multifactor phenomenon [3]. These metabolites are usually subdivided into field mycotoxins, produced on cereal crops before or immediately after harvest mainly by *Fusarium* spp., and storage mycotoxins, primarily secreted by *Aspergillus* and *Penicillium* spp. during commodity drying and storage [1].

Mycotoxin ingestion may induce various chronic and acute effects on humans and animals, such as hepatotoxic, genotoxic, immunosuppressive, estrogenic, nephrotoxic, teratogenic, and/or carcinogenic effects [1,4]. Moreover, mycotoxins are not completely eliminated during food processing operations and can contaminate finished processed food products [5,6].

Their worldwide occurrence in various food and feeds poses a major risk for human and animal health and, as a consequence, causes economic losses [1]. Although these economic costs are impossible to estimate accurately, the United States Food and Drug Administration (FDA) evaluated, based on computer modeling, that in the USA the potential economic costs of crop losses due to mycotoxin contaminations average $932 million per year [7]. The FAO (Food and Agriculture Organization of the United Nations) estimated that 25% of the world’s crops are affected by mycotoxins each year, with annual losses of around 1 billion metric tons of food and food products (2007).

Among the thousands of fungal secondary metabolites currently known, only a few groups of mycotoxins are important from the safety and economic points of view; namely aflatoxins (AFs), mainly produced by *Aspergillus* species; ochratoxin A (OTA), produced by *Aspergillus* and *Penicillium* species, and zearalenone (ZEA), fumonisins (FUM) and trichothecenes (TCTs) (especially deoxynivalenol (DON)), primarily produced by many *Fusarium* species [8,9,10]. Moreover, several species from the *Fusarium* genus can produce other mycotoxins with toxicological properties such as beauvericin (BEA), enniatins (ENNs), and moniliformin (MON), a group of lesser-studied toxins called emerging mycotoxins [11] (a non-exhaustive list of mycotoxin producing *Aspergillus*, *Penicillium* and *Fusarium* species, split into eight groups, is provided in Table 1). Even if these mycotoxin-producing fungi differ according to ecological conditions, it is important to emphasize that mycotoxins are found all over the world in foodstuffs and feedstuffs due to trade in these commodities that contributes to their worldwide dispersal. Moreover, Table 1 shows that one mycotoxin can be produced by several fungi, and that a fungus can produce several mycotoxins.

Several authors have shown an interest in cellular mechanisms and cellular toxicity in response to mycotoxin exposure. After ingestion by the consumer, the intestinal epithelium is the first host defense barrier against mycotoxins. However, although these cells are the first to be exposed to mycotoxins and at higher doses than other tissue cells, studies on the effect of mycotoxin mixtures on the gastrointestinal tract are scarce. Grenier and Appelgate [12] summarized in a recent review findings following major mycotoxin exposure (AFs, OTA, DON, T2, ZEA, and FUM) on digestive and absorptive functions, intestinal defense and microbiome composition. Briefly, they highlighted the large variability of mycotoxin bioavailability according to the considered mycotoxins and animal species. For example, the authors reported that more than 80% of AFs are absorbed within the gastrointestinal tract regardless of the non-ruminant species (*via* passive transport), whereas absorption of other major mycotoxins (TCT, OTA, or FUM) may vary from 1% to 60% (*via* passive transport by simple diffusion for OTA or *via* the paracellular route for DON). Moreover, several mycotoxins have been shown to undergo entero-hepatic circulation. This makes the mycotoxins available again *via* the bile in the entero-hepatic cycle, resulting in reabsorption and a prolonged retention time in the gastrointestinal tract. Intestinal metabolism in the gut epithelium and by the gut microbial population, limits the toxic effects of mycotoxins within the gastrointestinal tract. In particular, due to rumen microorganisms, ruminants are able to convert many mycotoxins into non-toxic metabolites before absorption, whereas for monogastrics, mycotoxin intestinal biotransformation takes place predominantly in the large intestine and thus provides little detoxification prior to absorption. However, little is known about the intestinal absorption and bacterial metabolism of the metabolites. Nevertheless, a recent *in vitro* study showed that the derivative 15-ADON caused the highest paracellular permeability and chemokine secretion compared to DON and 3-ADON in human intestinal cells Caco-2 [13]. Even if commensal microbiota is a key player in the detoxification against mycotoxins and their derivatives, it is important to note the potential of mycotoxins to enhance the toxic effects of intestinal pathogens and to change the intestinal microbiota balance by increasing the number of aerobic bacteria and thereby acting as a potential risk factor for chronic inflammatory diseases [12].

Because of their occurrence and toxicity, major mycotoxins (*i.e.*, AFs, OTA, ZEA, FUM, and DON) are the focus of legal regulations or guidance in many countries. The Joint Expert Committee on Food Additives (JECFA), a scientific advisory body of the World Health Organization (WHO) and the FAO, evaluates mycotoxin risks. In the United States and the European Union, regulatory and recommended guidance for mycotoxins are issued by the FDA and the European Commission (EC) advised by the European Food Safety Authority (EFSA), respectively. To protect animal and human consumers, these regulations fixed regulatory threshold values in food and feed to ensure they are not harmful and recommended good agricultural practice. For example, the maximum levels (MLs) of EU regulatory limits range from 0.1 µg/kg for aflatoxin B1 (AFB1) in processed cereal-based foods for human infants and young children, to 4000 µg/kg for fumonisins B1 and B2 in unprocessed maize for human consumption. Concerning milk and milk-based products, MLs are 0.05 µg/kg for aflatoxin M1 (European Commission (EC) 2006 and subsequent amendments) [14]. Mycotoxin regulations differ across states, even if harmonization efforts are being undertaken in some trade zones. However, this harmonization would not necessarily be beneficial from a human health protection point of view because of the differences in contamination levels and dietary habits in various parts of the world [15]. In the developed world, the dietary exposure is below the mycotoxin tolerance limits and tolerable daily intakes established by the JECFA, but it is not always the case for developing countries, as reported by Shepard [16], with the example of maize-based diet. Moreover, with the intensive farming due to an increasing world population, and particularly in developing countries, the number of world inhabitants over-exposed to mycotoxins could be enhanced over the next few years.

Noteworthy, mycotoxins may occur in modified forms from their parent compounds, due mainly to plant detoxification systems. Indeed, as part of their defense against xenobiotics, plants can alter the chemical structure of mycotoxins by modifications generated by enzymes involved in detoxification processes. Because these modifications lead to modified chromatographic profiles, epitope conformation or polarity, these mycotoxin derivatives usually escape conventional analytical methods and are not regulated by legislation and thus are called “masked” mycotoxins. Even if in the case of several studied conjugated mycotoxins, a lower toxicity was observed compared to the parent compounds, a potential increased bioavailability during digestion of masked mycotoxins still represent a health threat [17,18]. As masked mycotoxins are an emerging issue and insufficient toxicological and quantification data are available, these metabolites will not be considered in this review.

Concerning the routinely screened mycotoxins, the current regulations were established on toxicological data from studies taking into account only one mycotoxin exposure at a time, and do not consider the combined effects of mycotoxins. However, the natural co-occurrence of mycotoxins in cereals grains is well-known, and can be explained by at least three reasons: (i) most fungi are able to produce several mycotoxins concurrently (Table 1); (ii) food commodities can be contaminated by several fungi simultaneously or in quick succession and (iii) animal diets are usually made up of multiple grain sources. This is supported by a three-year worldwide survey that indicates that 48% of 7049 analyzed feedstuffs samples were contaminated by two or more mycotoxins [19]. This multi-contamination risk exposure is particularly true for ruminants, which have varied diets compared to other farm animals. In particular, ruminants are fed with forages, which are commonly contaminated with several mycotoxins, as reported in the recent review from Gallo *et al.* [20]. These authors highlighted the lack of data concerning mycotoxin occurrence in silages and other forage crops, and recommended to analyze forages for nutritive and fermentative characteristics, but also mainly for mycotoxin contaminations.

The toxicity of mycotoxins combinations cannot always be predicted based upon their individual toxicities. Multi-exposure may lead to additive, synergistic or antagonistic toxic effects [6,21]. The data on combined toxic effects of mycotoxins are limited, thus the health risk from this multi-exposure is not well-known.

The aims of this present review are to display the main natural mycotoxin mixtures found in common foods, such as cereals, nuts, fruits, milk and processed products thereof, and feedstuffs, to summarize current regulations as well as the published experiments on these mycotoxin mixtures, and to describe their known toxicological effects. This work may potentially underline areas lacking data for better taking into consideration this problem.

## 2. Mycotoxin Regulations

The last survey of the FAO in 2003 reported that, on a worldwide basis, around 100 countries, representing approximatively 87% of the world population, had regulations or detailed guidelines for mycotoxins or groups of mycotoxins in food and/or feed. Because of the various factors playing a role in the decision-making process to establish mycotoxin limits, including scientific, economic and political factors, the permitted limits and the mycotoxins targeted by legislation vary from country to country [15]. For example, the European Commission (EC) has issued maximum permitted levels for six groups of mycotoxins for animal feed: AFs, OTA, ZEA, FUM, DON, and rye ergot, and seven groups for human food: AFs, OTA, ZEA, FUM, DON, patulin (PAT) and citrinin (CIT); whereas only three groups are regulated by the FDA for animal feed (AFs, FUM, and DON) and one more for human food (PAT).

### 2.1. Aflatoxins

Regarding total aflatoxins (*i.e.*, sum of AFB1, AFB2, AFG1, and AFG2) in human food, EU MLs are 4 µg/kg for peanuts and other oilseeds, tree nuts, dried fruits, cereals, and processed products thereof, intended for direct human consumption or use as ingredient in foodstuffs; 10 µg/kg for tree nuts, dried fruits, maize and rice subjected to sorting, or other physical treatment, before human consumption as well as spices, dried figs, almonds, pistachios, apricot kernels, hazelnuts, and Brazil nuts intended for direct human consumption; and 15 µg/kg for peanuts and other oilseeds, almonds, pistachios, apricot kernels, hazelnuts, and Brazil nuts subjected to sorting, or other physical treatment, before human consumption [14]. The FDA action level is 20 µg/kg for total AFs in peanuts, Brazil nuts, pistachios, and other foods for direct human consumption [22].

Regarding animal feed, EU MLs have been issued for aflatoxin B1 only and range from 20 µg/kg for feed materials to 10 µg/kg for complementary and complete feed, with the exception of compound feed for young animals (MLs are 5 µg/kg) [23]. In comparison, the action levels established by the FDA for AFs range from 20 µg/kg for corn, peanut products, and other animal feeds and ingredients for immature and dairy animals, to 100 µg/kg for corn and peanut products for breeding cattle, breeding swine and immature poultry, 200 µg/kg for finishing swine and 300 µg/kg for finishing beef cattle as well as cottonseed meal for beef, cattle, swine or poultry, regardless of age or breeding status [22].

For milk and milk-based products, only aflatoxin M1 is considered, and EU MLs are 0.05 µg/kg [14]. Indeed, AFM1 is metabolized and excreted in the milk after the ingestion of its parent molecule, AFB1, by dairy cattle. The action levels established by the FDA are 10 times higher than the EU MLs for AFM1 in milk (namely 0.5 µg/kg) [22].

### 2.2. Ochratoxin A

OTA MLs in the EU are 0.5 µg/kg for processed cereal-based foods and baby foods; 2 µg/kg for wine, grape juice, grape nectar and grape must intended for direct human consumption; 3 µg/kg for products derived from unprocessed cereals; 5 µg/kg for unprocessed cereal, roasted coffee beans and ground roasted coffee; 10 µg/kg for dried vine fruit and soluble coffee; 15 µg/kg for certain spices; 20 µg/kg for liquorice root for herbal infusion and 80 µg/kg for liquorice extract for use in food in particular beverages and confectionery [14].

For animal consumption, MLs are 250 µg/kg for feed materials, 50 µg/kg for complementary and complete feeding stuffs for pigs, and 100 µg/kg for poultry [33].

The FDA does not establish regulatory guidance for this toxin.

### 2.3. Fumonisins

Concerning FUM, the EC has set MLs for the sum of fumonisins B1 and B2, ranging from 200 µg/kg for processed cereal-based and baby foods for infants and young children, to 4000 µg/kg for unprocessed maize. FUM may also be found in other common foods such as maize and maize-based foods intended for direct human consumption (MLs are 1000 µg/kg), or maize-based breakfast cereals and snacks (MLs are 800 µg/kg) [14]. The FDA guidance levels for the sum of fumonisins B1, B2, and B3 are between 2000 µg/kg and 4000 µg/kg for maize and maize-based products intended for human food [34].

Moreover, MLs for the sum of FB1 and FB2 are 60,000 µg/kg for maize and maize products in feed materials and range from 5000 µg/kg to 50,000 µg/kg for complementary and complete feeding stuffs, depending on the species and the age of the animal (MLs are 5000 µg/kg for pigs, equids, rabbits and pet animals, 10,000 µg/kg for poultry, calves, lambs and kids, and 50,000 µg/kg for adult ruminants and mink) [33]. The FDA guidance levels for the sum of FB1, FB2, and FB3 range from 5000 µg/kg to 100,000 µg/kg for corn and corn by-products in animal feed according to species and age (FDA guidance levels are 5000 µg/kg for equids and rabbits, 20,000 µg/kg for swine and catfish, 30,000 µg/kg for breeding ruminants, poultry and mink, 60,000 µg/kg for ruminants being raised for slaughter and mink being raised for pelt production, 100,000 µg/kg for poultry being raised for slaughter, and 10,000 µg/kg for all other species and classes of livestock) [34].

### 2.4. Zearalenone

EU MLs for ZEA in human food are 20 µg/kg for processed maize-based foods for infants and young children, and processed cereal-based foods; 50 µg/kg for bread, pastries, biscuits, cereal snacks and breakfast cereals; 75 µg/kg for cereals intended for direct human consumption; 100 µg/kg for maize, maize-based snacks, maize-based breakfast cereals and unprocessed cereals; 350 µg/kg unprocessed maize and 400 µg/kg for refined maize oil [14]. The FDA does not establish regulatory guidance for this toxin.

For feed materials, MLs range from 2000 µg/kg for cereals and cereal products, to 3000 µg/kg for maize products. Concerning complementary and complete feeding stuffs, MLs range from 100 µg/kg for piglets and young sows, to 250 µg/kg for sows and fattening pigs and 500 µg/kg for calves, dairy cattle, sheep, and goats [33].

### 2.5. Trichothecenes

Regarding DON in human food, MLs range from 200 µg/kg for processed cereal-based and baby foods to 1750 and 1250 µg/kg for unprocessed durum wheat, oats, and maize as well as other unprocessed cereals, respectively. DON may also be found in other common foods such as cereals intended for direct human consumption and pasta (in this case MLs are 750 µg/kg), as well as bread, pastries, biscuits, cereal snacks, and breakfast cereals (MLs are 500 µg/kg) [14]. The FDA advisory level for DON is 1000 µg/kg for finished wheat products intended for direct human consumption [35], and thus is close to the EU MLs. Currently, levels are under discussion for the sum of T-2 and HT-2 toxins in unprocessed cereals and cereals products for human consumption in the EU [14].

For feed materials, the EU MLs range from 8000 µg/kg for cereals and cereal products, to 12,000 µg/kg for maize by-products. EU MLs for complementary and complete feeding stuffs are 5000 µg/kg expect for pigs (MLs are 900 µg/kg) and calves, lambs and kids (MLs are 2000 µg/kg) [33]. For grain and grain by-products in animal feed, the FDA advisory levels range from 5000 µg/kg to 10,000 µg/kg (according to the considered species and the age of the animal) [35], whereas due to the relatively low human exposure to the other TCTs, such as nivalenol (NIV) and diacetoxyscirpenol (DAS), and their co-occurrence with typically more abundant DON, establishing maximum permitted levels for these toxins is currently not considered [14]. However, due to their possible additive or synergistic toxic effects, it would be interesting to establish regulations for total TCTs, as it is already the case with AFs and FUM.

### 2.6. Other Regulated Mycotoxins

Regarding patulin (PAT), the EU MLs are 10 µg/kg for apple juice and solid apple products, including apple compote and apple purée, for infants and young children. Moreover, MLs are 25 µg/kg for solid apple products for direct human consumption and 50 µg/kg for fruit juices, spirit drinks, cider and other fermented drinks derived from apples or containing apple juice [14]. FDA regulatory limits are 50 µg/kg for apple juice and apple juice component of a food that contains apple juice as an ingredient [36].

EU MLs for citrinin (CIT) are 2000 µg/kg for food supplements based on rice fermented by the “red yeast” *Monascus purpureus* [14].

MLs for rye ergot in the EU are 1000 mg/kg for feed materials and compound feed containing ungrounded cereals [33].

Thus, the European Community has one of the most stringent regulations in the world, with numerous mycotoxins and commodities concerned, and more restrictive levels. However, like the other regulations in the world, the EC does not consider the combined toxicological effects of mycotoxins.

## 3. Natural Co-Occurrence of Mycotoxins in Foods and Feeds

Several surveys reported the natural co-occurrence of mycotoxins from all over the world, and most of them concerned the major mycotoxins AFs, OTA, ZEA, FUM, and TCTs—especially DON. However, only a few studies specified the number of co-occurring mycotoxins with the percentage of the co-contaminated samples, as well as the main combinations found. We selected the relevant data and papers (from 1987 to present) from over a hundred papers dealing with mycotoxin co-occurrence in different foods and feeds. Only studies with at least 10 samples were considered.

**Figure 1:**
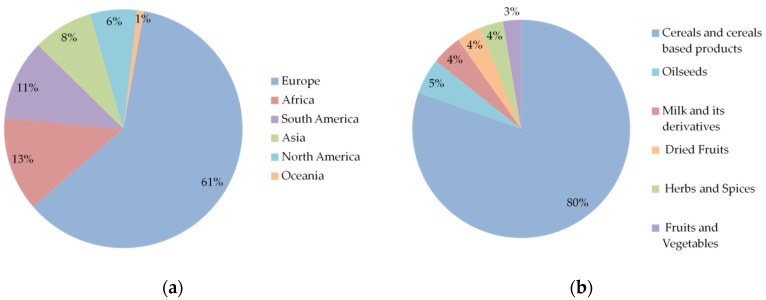
Data distribution depending on (**a**) geographic regions and (**b**) commodities. Data compiled from 107 articles. References: [37,38,39,40,41,42,43,44,45,46,47,48,49,50,51,52,53,54,55,56,57,58,59,60,61,62,63,64,65,66,67,68,69,70,71,72,73,74,75,76,77,78,79,80,81,82,83,84,85,86,87,88,89,90,91,92,93,94,95,96,97,98,99,100,101,102,103,104,105,106,107,108,109,110,111,112,113,114,115,116,117,118,119,120,121,122,123,124,125,126,127,128,129,130,131,132,133,134,135,136,137,138,139,140,141,142,143].

As presented in Figure 1a, more than 60% of the information comes from Europe, whereas merely 7% is obtained from North America, and only one paper studied samples from Oceania. Concerning the commodity types, raw and processed cereals are the most frequently studied, representing 80% of the overall data. The rest of the data mainly concerns plant products, especially fruits, spices, and nuts, and only a few studies were focused on milk and its derivatives (Figure 1b). Overall, about 50% of the data concerning cereals and cereal based-products comes from Europe (data not shown). Additionally, amongst the 107 included studies [37,38,39,40,41,42,43,44,45,46,47,48,49,50,51,52,53,54,55,56,57,58,59,60,61,62,63,64,65,66,67,68,69,70,71,72,73,74,75,76,77,78,79,80,81,82,83,84,85,86,87,88,89,90,91,92,93,94,95,96,97,98,99,100,101,102,103,104,105,106,107,108,109,110,111,112,113,114,115,116,117,118,119,120,121,122,123,124,125,126,127,128,129,130,131,132,133,134,135,136,137,138,139,140,141,142,143], about 35% was published between 2011 and 2015, highlighting the increasing interest for worldwide mycotoxin co-occurrence.

The difficulty of comparing studies using different methodologies of mycotoxin detection and quantification should be emphasized, considering their associated sensitivity and accuracy variations. Indeed, since 1972, we have witnessed a tremendous evolution of chromatographic and immuno-techniques. Especially since 2011 with the development of LC or GC-MS/MS that can detect ever more co-occurring mycotoxins. Moreover, some authors focused on only certain mycotoxins while others developed non-targeted approaches, which also complicates qualitative and quantitative comparisons. For example, the last worldwide mycotoxin survey [144] found up to 75 co-occurring mycotoxins in a same sample from a LC-MS/MS analysis targeting more than 380 mycotoxins simultaneously, whereas up to seven co-occurring mycotoxins were found in a same sample among the 107 papers analyzed, with a more “classic” approach targeting less than 15 major mycotoxins [123].

The main mixtures reported in these articles were analyzed by commodity type (cereals and cereals based-products, herbs and spices, dried fruits, fruits and vegetables, oilseeds, and milk and its derivatives) and by region (Europe, Africa, Asia, South America, and North America). Because only one study cites a sample coming from New Zealand, Oceania was not included.

### 3.1. Results by Commodity Type

Among the 116 mycotoxin combinations found by the authors in cereal and derived cereal product samples, AFs+FUM, DON+ZEA, AFs+OTA, and FUM+ZEA were the most present. These mixtures are quoted 21, 14, 12, and 11 times out of the 91 papers analyzing cereal products, respectively, representing 23%, 15%, 13%, and 12% of these articles respectively. Furthermore, the last survey by the BIOMIN Company showed that DON, FUM, and ZEA are the most prevalent mycotoxins in the world, with a prevalence of 66%, 56%, and 53%, respectively, among the 6844 analyzed agricultural commodity samples [144]. Because of their common co-occurrence, also potentially associated with AFs (with a worldwide prevalence of 22%) [144], these mycotoxin toxicological interactions must not be disregarded.

Only four papers focused on herbs and spices [68,75,110,122]. In all of them, AFs+OTA mixtures were listed. The other combinations found corresponded to OTA+ZEA, AFs+ZEA, and AFs+OTA+ZEA, quoted twice for OTA+ZEA and AFs+OTA+ZEA, and once for AFs+ZEA.

Dried fruits were also studied in four papers [45,68,74,127]. In this context, the AFs+OTA mixture was cited three times and AFs + cyclopiazonic acid (CPA) only once.

Among the three articles concerning fruits and vegetables, apples have been extensively studied [73,101,124]. Five mycotoxin mixtures were reported in these articles and none of the authors found the same mixtures. It should be noted that PAT was quoted twice in combination with either AFs or CIT.

The same observation was made for oilseeds (nuts, tree nuts, soy, olives): among the 11 mixtures quoted in six papers, all are cited only once [59,60,63,120,122,123]. The combinations listed were mainly formed with TCTs.

Concerning milk and its derivatives, mainly cheeses, only three mixtures have been reported: Roquefortine-C (ROQ-C) + mycophenolic acid (MYC-A), AFs+OTA, and AFs+CPA. These combinations were quoted 2-, 2- and 1-times out of five articles, respectively [42,66,86,91,108]. Other animal products, like meat or eggs, have not been studied in a co-occurrence context.

Using this literature set, it can be summarized that AFs are found in various food and feed products, often in combination with OTA or fusariotoxins (mainly FUM and ZEA). Generally, binary mixtures are the most common among about 25 mycotoxins studied in the 107 papers, even if the last BIOMIN survey showed, that among the worldwide samples tested on average 30 different metabolites were detected per sample using a multi-mycotoxin technique (LC-MS-MS) [144].

### 3.2. Results by Region

**Figure 2:**
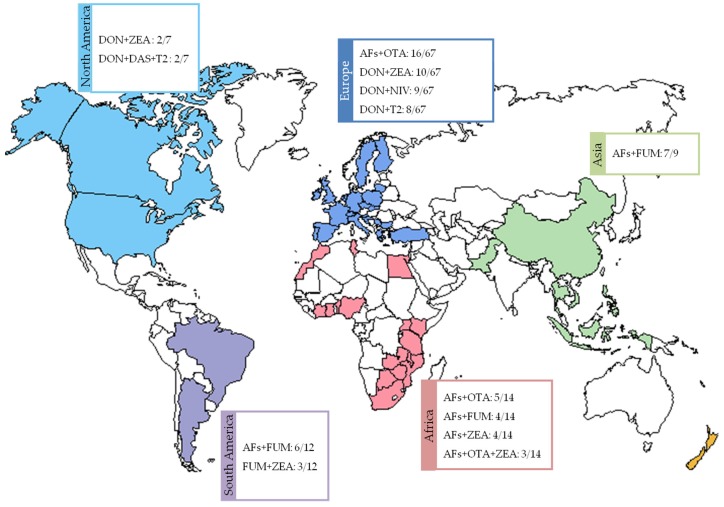
Main mycotoxin mixtures quoted in the papers depending on their geographic origin. Data compiled from 107 articles. References: [37,38,39,40,41,42,43,44,45,46,47,48,49,50,51,52,53,54,55,56,57,58,59,60,61,62,63,64,65,66,67,68,69,70,71,72,73,74,75,76,77,78,79,80,81,82,83,84,85,86,87,88,89,90,91,92,93,94,95,96,97,98,99,100,101,102,103,104,105,106,107,108,109,110,111,112,113,114,115,116,117,118,119,120,121,122,123,124,125,126,127,128,129,130,131,132,133,134,135,136,137,138,139,140,141,142,143].

The relation between geographical origin and reported mycotoxin combinations is presented in Figure 2. For European samples, among the 105 mycotoxin mixtures found, the most reported one (16 out of 67 publications, or 24%) was AFs+OTA. While, DON+ZEA, DON+NIV, and DON+T2 combinations were quoted in 15%, 13%, and 12% of these articles, respectively. The other combinations were listed in less than 10% of the articles.

Concerning African samples, over the 26 observed mycotoxins combinations, AFs+OTA was once again, the main mixture, representing 35% of the 14 publications related to African samples. The AFs+FUM and AFs+ZEA binary combinations as well as the AFs+OTA+ZEA ternary combination were cited in 29%, 21%, and 29% of these articles, respectively. The other mixtures were observed in only two or less articles.

In Asia, AFs+FUM was the most observed mixture (seven out of nine articles, or 78%) among the 18 listed combinations. The other combinations were reported in only one or two articles. It can be highlighted that AFs or FUM were present in almost all the other mixtures.

In South America, more particularly in Brazil and Argentina, AFs+FUM was also the most observed mixture, as it was reported in 50% (six out of 12 articles). While FUM+ZEA was the second most observed combination (25%) among the 12 listed mycotoxins mixtures.

Concerning the seven publications from North America, 21 mycotoxin combinations were reported, the main ones being DON+ZEA and DON+DAS+T2, quoted in two papers (29%), respectively.

In conclusion regarding the occurrence and prevalence aspect, the AFs+FUM mixture is the most prevalent in Africa, Asia, and South America (Figure 2). Maize harvested in the tropical and subtropical areas of the world with hot and humid climates is the major commodity contaminated with the two toxins. Aflatoxins are a far greater problem in the tropics than in temperate zones of the world. However, because of the movement of agricultural commodities around the globe, no region of the world is aflatoxin-free. In more temperate and cold regions (Europe and North America), mixture of TCTs or TCTs with ZEA are the most common, highlighting the importance of the climate conditions on fungal contamination, growth, metabolism and thus mycotoxin mixtures. *Fusarium* is the main genus implicated in TCTs production and many toxigenic *Fusarium* species have been associated with infected grain. The geographical distribution of the *Fusarium* species is probably related to environmental temperature requirements and/or different agricultural practices [145].

Overall, among the 127 mycotoxin mixtures described by the authors from all combined countries and commodities, the main mycotoxin mixtures cited were AFs+OTA, AFs+FUM, and DON+ZEA, found in 21%, 20%, and 13% of the studies. Cereals represent the main OTA and ZEA sources of human intake [146,147]. Among cereal grains, AFs and ZEA mainly appear in corn (EFSA, 2004; EFSA 2007), whereas barley has a particularly high likelihood of OTA contamination [148]. Over the past few years, there has been emerging evidence of potential aflatoxin contamination of feed materials grown in areas of southern Europe, where a subtropical climate and extensive agricultural practice favor fungal growth and the subsequent formation of aflatoxins (EFSA, 2007). However, it is important to note that our analysis did not consider the “year” parameter, and it is well known today that prevalence and contamination levels of mycotoxins vary greatly according to harvest year of the cereals [149]. Moreover, climatic and agricultural practice changes observed over the last years, including the reduction of fungicide use, could lead to mycotoxin contamination in food [150,151].

**Figure 3:**
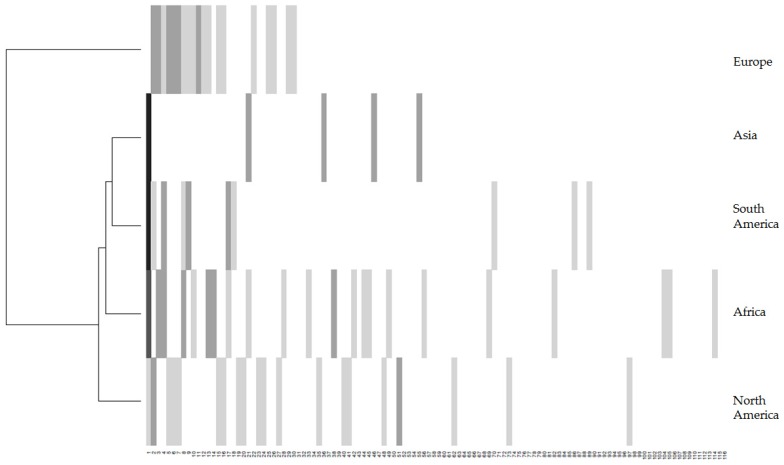
All mycotoxin mixtures quoted in the papers depending on their geographic origin. (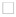 = mixtures no cited; 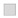 = mixtures cited between 1 and 3 times; 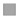 = mixtures cited between 3 and 5 times; 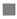 = mixtures cited between 5 and 7 times; 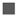 = mixtures cited between 7 and 9 times; 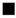 = mixtures cited more than 9 times). Reading from left to right on the *x*-axis:

Based on the data organized by region, a dendogram was created using the “HeatMap” function of the “R Project for Statistical Computing” software and a hierarchical ascendant classification analysis using the “hclust” function and with the default parameter “ward’s method”. This graphic representation, corresponding to a qualitative approach, is a heat-grey plot matrix illustration, in which the grey color intensity depends on the number of times that a mycotoxin combination mixture is cited (Figure 3). Asia and South America exhibit similar profiles; they are as close to Africa’s profile as the same mixtures, with a similar number of reports, have been observed. Despite the fact that EU regulations are one of the most stringent in the world, Europe exhibits a large range of mixtures cited compared to the other regions but it is worth nothing that European studies were more extensive as they represent 61% of the 106 studied articles. Thus, the significant difference in the number of publications by region could also impact on the results. Nevertheless, North America has the closest profile to Europe. This analysis was supported by the above comments which highlighted the role of climate in mixture occurrence and potentially by similar agricultural methods.

1. AFs FUM41. DAS HT281. BEA ENNs MON2. DON ZEA42. T2 NIV82. AFs OTA DON3. AFs OTA43. T2 ZEA83. AFs OTA T24. FUM ZEA44. NIV BEA84. AFs OTA NIV5. DON NIV45. ENNs FUS85. AFs OTA FUM6. DON T246. AFs DON86. AFs FUM T27. DON HT247. FUM BEA87. AFs DON ZEA8. AFs ZEA48. FUM MON88. FUM DON NIV9. FUM DON49. OTA NIV89. FUM T2 HT210. FUM OTA50. DON ADON T290. FUM BEA OTA11. DON T2 ZEA51. DON DAS HT291. FUM BEA FUS12. T2 HT252. DON T2 NIV92. FUM OTA CIT13. BEA ENNs53. DON HT2 FUS-X93. DON ADON T2 HT214. AFs OTA ZEA54. DON OTA ZEA94. DON ADON HT2 ZEA15. DON ADON NIV55. AFs FUM NIV95. DON ADON ZEA αZOL16. DON ADON ZEA56. AFs BEA NIV96. DON MAS NIV ZEA17. AFs FUM ZEA57. DON ADON T2 NIV97. DON DAS T2 HT218. FUM DON ZEA58. DON ADON T2 ZEA98. DON T2 ZEA αZOL19. DON ADON59. DON T2 HT2 NIV99. DON HT2 NIV ZEA20. DON OTA60. DON T2 NIV ZEA100. DAS T2 HT2 ZEA21. FUM NIV61. DON ADON T2 HT2 ZEA101. T2 HT2 MAS ZEA22. OTA ZEA62. DON DAS102. T2 HT2 NIV BEA23. OTA CIT63. DON αZOL103. NIV ZEA BEA ENNs24. DON T2 HT264. DON ENNs104. AFs OTA DON ZEA25. DON HT2 NIV65. NIV HT2105. AFs OTA T2 ZEA26. DON HT2 ZEA66. NIV FUS-X106. FUM DON NIV ZEA27. DON NIV ZEA67. NIV ZEA107. DON ADON MAS HT2 ZEA28. FUM ZEA OTA68. ZEA ENNs108. DON ADON HT2 NIV ZEA29. DON ADON HT2 NIV69. BEA FUS109. DON ADON HT2 NIV FUS-X30. DON T2 HT2 ZEA70. FUM T2110. DON DAS T2 HT2 ZEA31. DON FUS-X71. FUM FUS111. DON MAS T2 HT2 ZEA32. ADON ZEA72. MYC-A ROQ-C112. DON T2 HT2 NIV ZEA33. AFs NIV73. DON DAS T2113. MAS T2 HT2 NIV ZEA34. DON ADON HT274. DON NIV FUS-X114. AFs FUM OTA DON ZEA35. DAS T2 HT275. DON NIV MAS115. DON ADON T2 NIV ZEA αZOL36. AFs FUM DON76. DON ZEA αZOL116. DON ADON NIV ZEA αZOL βZOL37. DON ADON NIV ZEA77. T2 T2tetraol HT238. AFs FUM OTA ZEA78. T2 HT2 ZEA39. DON ADON T2 HT2 NIV79. NIV FUS-X BEA40. DAS T280. BEA ENNs FUS

## 4. Toxicological Impact of Mycotoxin Interactions

As stated previously, toxicological evaluation and therefore regulations are based so far on individual mycotoxin. However, as confirmed by the data analysis presented in the first part of this review, single mycotoxin contamination is not the norm but rather the exception. It is therefore of the utmost importance to evaluate the toxicological impact of mycotoxin combinations to better reflect feed and food contamination and their associated animal and human health risks. In this context, Grenier and Oswald [6] reviewed *in vivo* experiments until 2010, in which laboratory and farm animals were exposed to a combination of mycotoxins, and described the type of observed interactions. Since 2011, only few *in vivo* studies have been published. In the framework of this review, we focused on *in vitro* experiments published between 1980 and 2015. Indeed, even if cell cultures have many limitations such as immortalization, limited survival or metabolic imbalance, *in vitro* models are more and more used for understanding the mechanisms of mycotoxin action and their mixtures, especially toxicity on cell-specific function [152]. Among the 58 analyzed articles, 50% were published during the last five years showing the interest of this approach as an alternative of interest to animal models. In this context, *in vitro* studies become embedded in national and international legislation regulating the use of animals in scientific procedures in order to encourage and develop the principles of the 3Rs (Replacement, Reduction, and Refinement) as a framework for humane animal research.

Most of the selected publications concern the effect of binary mixtures. Indeed, among the 93 studied mycotoxin mixtures, 70% corresponded to binary mixtures, 24% to ternary mixtures, and 6% were quaternary or quinary mixtures. Furthermore, the main studied mixtures were OTA+CIT, DON+NIV, DON+T2, OTA+AFB1, and OTA+FB1 found in 28%, 14%, 12%, 10%, and 9% of the articles, respectively. Another observation corresponds to the fact that mixtures involving fusariotoxins were the most studied, representing about 70% of all the analyzed mixtures, with 50% involving exclusively fusariotoxins and 22% are formed with OTA.

**Figure 4:**
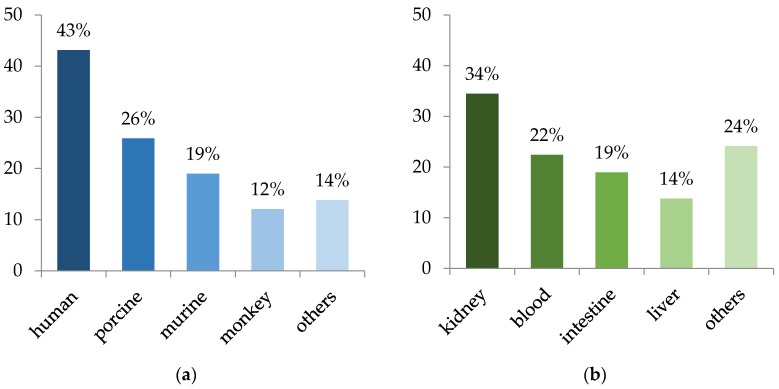
Distribution of cell models used depending on (**a**) species and (**b**) organs. Data are compiled from the 58 selected articles. References: [9,154,155,156,157,158,159,160,161,162,163,164,165,166,167,168,169,170,171,172,173,174,175,176,177,178,179,180,181,182,183,184,185,186,187,188,189,190,191,192,193,194,195,196,197,198,199,200,201,202,203,204,205,206,207,208,209,210].

Concerning cell models, 43% of the authors used, *inter alia*, cells from human origin, 26% porcine models, 19% murine models, and more marginally monkey, bovine, fish, turkey or/and even yeast, which is a simple model to examine the immediate effects of mycotoxins on growth inhibition or CO_2_ production for example (Figure 4a). Overall, more than 30 different cell lines were used among the 58 articles studied, and most of these cells came from kidney, blood, intestine, and liver (Figure 4b). More particularly, Caco-2 (human epithelial colorectal adenocarcinoma cells), PK15 no copyright permission needed as we created this figure (porcine hepatocellular carcinoma cells), Vero (monkey renal proximal tubular epithelial cells), and HepG2 (human kidney epithelial cells) were the most used cell models as they were reported in 8, 8, 7, and 5 articles, respectively. This is linked to the fact that these cell models correspond to major organs targeted by mycotoxins [153].

Regarding the studied parameters, cell viability was the main endpoint used by the authors (in 64% of the studies), followed by cell apoptosis or/and necrosis (19%), DNA damage (17%) and oxidative damage (16%). Some authors were also interested in macromolecule synthesis (RNA, DNA, proteins), or immunotoxicity parameters. Moreover, all these tests are performed between 0 and 72 h (acute exposure), except in the work of Ficheux *et al.* [166], in which mycotoxin interactions were studied during 14 days (chronic exposure). In particular, for cell viability, studies were mostly carried out on 24 h and/or 48 h, with the most commonly used being the tetrazolium reduction assays. Different tetrazolium reduction assays exist, based on similar principles, such as MTT 3-(4,5-dimethylthiazol-2-yl)-2,5-diphenyltetrazolium bromide, MTS 3-[4,5-dimethylthiazol-2-yl]-5-(3-carboxymethoxyphenyl)-2-(4-sulfophenyl)-2*H*-tetrazolium and WST-1 (2-(4-iodophenyl)-3-(4-nitrophenyl)-5-(2,4-disulfophenyl)-2*H*-tetrazolium). The neutral red and trypan blue assays are two other methods commonly used to evaluate cell viability. Some authors assessed mycotoxin toxicological effects individually and/or combined on cell proliferation using two or three cell viability assays (tetrazolium reduction, neutral red and trypan blue assays) and results were similar from one method to the other [9,155,160,185,197].

In the present review, we decided to focus on the *in vitro* effects of fusariotoxin mixtures on cell viability using mammalian cell models (Table 2). Concerning mycotoxin mixtures involving OTA, a review about their *in vitro* and *in vivo* combined effects was recently published [211].

To better understand the conclusions presented by the authors about the *in vitro* effects of fusariotoxin mixtures, the main types of interactions between mycotoxins, as well as mathematical models for characterizing these interactions, are described hereafter.

### 4.1. Characterization of the Different Interactions Between Mycotoxins

**Figure 5:**
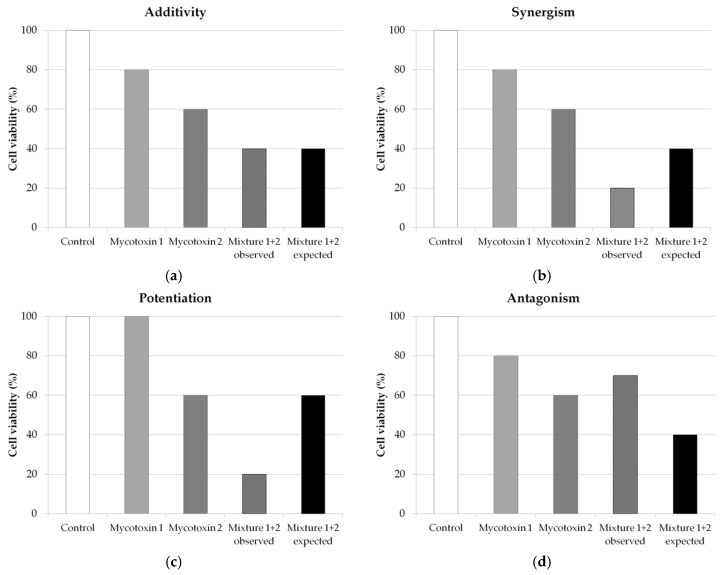
Characterization of the interaction between mycotoxins.

Mycotoxin interactions can be classified in three main different categories: antagonistic, additive, and synergistic. Depending on the authors, more categories may be distinguished, namely potentiation and less-than-additive, often classified in synergistic and antagonistic effects, respectively. Figure 5 illustrates the possible different interactions of mycotoxins with the example of cell viability measure.

Additivity is mentioned when the effect of the combination could be calculated as the sum of the individual effects of the two studied toxins (Figure 5a). Thus, additivity is *a priori* an absence of interaction.
Synergism is observed when the effect of the mycotoxin combination is greater than expected in comparison to the sum of the individual effects of the two studied mycotoxins (Figure 5b). In the case when one or both of the mycotoxins does not induce effect whereas the combination induces a significant effect, one can speak of potentiation (Figure 5c). However, very few studies use this term to categorize the effect, and most of them use synergism.
Antagonism is cited when the effect of the mycotoxin combination is lower than expected from the sum of the individual effects of the two studied mycotoxins (Figure 5d). If the effect of the mycotoxin combination mainly reflected the effect of the most toxic mycotoxin, without additional effect of the other mycotoxin, the term “less-than-additive” may be used.

A deeper view of the different interactions between mycotoxins can be found in the review by Grenier and Oswald [6], in which three types of synergism are presented and two kinds of antagonistic effects are itemized.

### 4.2. Main Experimental Designs for Studying Mycotoxin Interactions

Several experimental designs can be used for studying mycotoxin interactions. Klarić *et al.* [152] briefly described the main mathematical designs used for this purpose: central composite design (CCD), full factorial design, ray design, isobolographic analyses/combination index, and the arithmetic definition of additivity. Some authors used other approaches such as the interaction index V [159] and the coefficient of drug interaction (CDI) [210] to characterize the type of interaction. The aim of all these experimental designs is to predict combined mycotoxin effects based on the comparison between the observed and expected effects of a mycotoxin mixture. The most used models are described hereafter.

The main approach is the one applied by Weber *et al.* [212] and used in more than 30% of the 58 studies. This method is based on the comparison of theoretical expected values calculated on the basis of mono-exposure experiment results with the observed values obtained from co-exposure experiment. In the case of binary mycotoxin combination exposure, the expected cell viability value is calculated as follows:

Cell viability expected value for Mycotoxin1 + Mycotoxin2 (%) = mean cell viability for Mycotoxin1 (%) + mean cell viability for Mycotoxin2 (%) − mean control condition (100%)

The expected standard error of mean (S.E.M.) is calculated as follows:

S.E.M. expected for Mycotoxin1 + Mycotoxin2 = [(S.E.M. for Mycotoxin1)² + (S.E.M. for Mycotoxin2)²]^1/2^

Combined cytotoxic effects are determined by comparison between each expected value and the corresponding measured mean value obtained from co-exposure experiments, often using an unpaired *t*-test. No statistical difference between expected and measured cell viability values is interpreted as an additive effect on cell viability reduction, whereas a synergistic or antagonistic effects are determined if the measured cell viability values are respectively significantly below or above the expected values.

**Figure 6:**
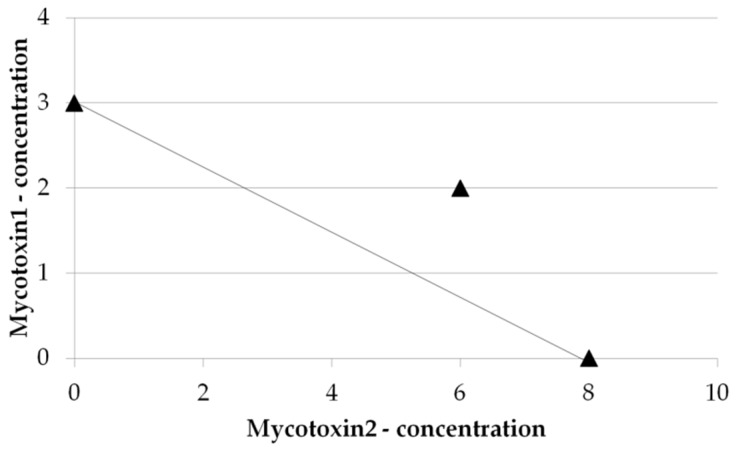
Isobologram illustrating the antagonist effect of two mycotoxins for reaching *x*% of cell viability inhibition.

The second most used method, applied in 22% of the analyzed articles, is the combination index-isobologram analysis also known as the Chou-Talalay method [213,214], derived from the Median-effect principle and originally used for analyzing drug combination effects. In isobolographic analyses, the isoeffective points can be interpolated from the results (of cell viability tests for example) and used to plot the isobologram, represented by a line joining equally effective doses (Figure 6). In this type of graph, the additive effect follows the diagonal line between the effective concentrations of each single mycotoxin. If the measured combined effect of two mycotoxins is above or below the diagonal line, it indicates an antagonist or a synergistic effect of the combination respectively. Chou introduced the term “combination index” (CI) to quantify the degree of mycotoxin interaction between two or more mycotoxins [213]. The CI method is often used to analyze the mycotoxin interaction, and the CI values are calculated as follows:

$${\left(\text{CI}\right)}_{x}^{n}=\;\overset{n}{\underset{j=1}{\sum }}\frac{{\left(D\right)}_{j}}{{\left(D_{x}\right)}_{j}}$$

where ${\left(\text{CI}\right)}_{x}^{n}$ is the CI for n mycotoxins at x% cell viability inhibition, ${\left(D\right)}_{j}$ is the doses of n toxins that exerts x% inhibition in combination, ${\left(D_{x}\right)}_{j}$ is the doses of each of n mycotoxins alone that exerts x% inhibition.

A CI near 1 indicates an additive effect, CI < 1 indicates synergism, and CI > 1 indicates antagonism of the combined mycotoxins. The CI-isobologram method allows not only for determination of the type of interaction but also of its magnitude. This is presented in more detail by Ruiz *et al.* [197] and others.

Only four authors used a CCD including a full or fractional factorial design for mixtures [173,188,202,209]. Briefly, the CCD is used in order to minimize the number of possible toxin combinations from all possible combinations of every concentration (*m* concentrations) of each toxin (*k* toxins) = *mk*, to *n* = 2*k*/2 cube points + 2*k* star points + 1 center point. Then, a full or fractional factorial design is applied to detect interactions at various mixture ratios [173]. Nevertheless, when the number of mycotoxins increases and the number of design points needed to study the toxin mixtures becomes too high, another alternative is the ray design providing constant mixture ratios and thus reducing the amount of experimental efforts. Only one author group out of the 58 analyzed articles used this design [203].

The sample number is not a limiting factor regardless of the considered model. However, the simplest and the most intuitive mathematical design seems to be the arithmetic definition of additivity and applied by Weber *et al.* [212] because it is based on a simple additivity of the individual mycotoxin toxicological effect values. Nevertheless, this definition of the combined effects, namely simply defined by the sum of single effects, is questionable, and the example of the combined effect study of the sum of several doses of the same mycotoxin, which cannot be synergistic or antagonistic, highlights this point.

It could be interesting to use different statistical models to analyze a specific mycotoxin mixture under identical exposure conditions to verify the similarity of the results and conclusions, and thus, to determine if it is necessary to standardize the method.

### 4.3. In Vitro Interactions Between Fusariotoxins

The global results from *in vitro* cell viability studies concerning fusariotoxin mixtures (subdivided in as follows: TCT mixtures; TCT + fusariotoxins and other fusariotoxins mixtures) are presented in Table 2. According to the analyzed studies, trichothecenes as well as other fusariotoxins (ZEA, FUM and emerging mycotoxins), individually and in combination, inhibit cell viability *in vitro*.

Alassane-Kpembi *et al.* [154,155] showed that combination of DON and its acetylated derivatives 3-DON and/or 15-ADON mainly resulted in synergistic cytotoxicity on porcine IPEC-1 and human Caco-2 cells, and particularly at low inhibitory concentration levels (Inhibitory Concentrations from 10% to 30% = IC_10_–IC_30_) on Caco-2. Additive effects were observed at higher doses (IC_50_). Concerning one of the most studied mixtures, DON+NIV, multiple effects have been observed. The same authors showed synergistic effects on Caco-2 and IPEC-1 (between 0.2 and 15 µM) [154,155], while Wan *et al.* observed antagonistic effect at 0.5 µM and synergism at 2 µM on porcine IPEC-J2 [209], whereas Marzocco *et al.* described additivity at medium cytotoxicity level (IC_50_, or 15 µM) on murine J774A.1 [194]. DON+FX resulted in synergistic cytotoxicity on Caco-2 and antagonistic effect on IPEC-1, whereas NIV+FX resulted in synergistic effect at low cytotoxicity levels (IC_10_–IC_20_) and additivity at higher inhibitory concentration levels (IC_30_–IC_50_) on Caco-2 and only additivity on IPEC-1 (IC_10_–IC_80_) [154,155]. For DON+T2, antagonism was observed with acute exposure (24 to 72 h) on Chinese hamster CHO-K1 and monkey Vero cells [197,198], and additivity was reported with human progenitors CFU-GM with 14 days of exposure [166]. Therefore, even if a global observation of synergistic toxicity was often observed at low cytotoxicity doses (IC_10_–IC_30_), trichothecene mixtures resulted in various cytotoxicity effects which seem to depend on the studied mycotoxin combination, the used cell model, the time of exposure and the tested concentration.

Several authors were interested in mixtures of TCT and other fusariotoxins, such as FB1, ZEA, and the emerging mycotoxin BEA. Again, the conclusions of the different authors and studies were species- and organ-dependent: Ruiz *et al.* observed antagonistic effects on hamster CHO-K1 and monkey Vero cells with DON+BEA co-exposure [197,198], whereas Ficheux *et al.* showed synergism on human CFU-GM [166]. Ruiz *et al.* also studied T2+BEA and showed opposite cytotoxic effect on CHO-K1 and Vero cells (synergism and antagonism respectively) despite the similar mycotoxin doses, the same time of exposure (24 to 72 h) and the same used assessment to measure cell viability (neutral red assay) [197,198]. These opposite observations highlight the complexity of the mycotoxin interactions, with the influence of the used cell models (studied species and targeted organs).

Regarding the ternary mixture DON+T2+BEA studied by Ruiz *et al.*, the effects were the same as those observed for T2+BEA on CHO-K1 and Vero cells [197,198]. Ficheux *et al.* [166] as well as Wan *et al.* [209] observed antagonistic effects with DON+FB1 on CFU-GM and IPEC-J2 at low concentrations, respectively (less than 0.5 µM DON and 20 µM FB1), whereas Kouadio *et al.* showed additivity on Caco-2 at similar doses [187]. Wan *et al.* also observed the same effect on IPEC-J2 with NIV+FB1 and DON+NIV+FB1, that DON+FB1 (namely antagonism at the lowest dose (0.5 µM DON and NIV, and 20 µM FB1) and synergism at the highest dose (2 µM DON and NIV, and 40 µM FB1)) [209]. Concerning DON+ZEA, another mixture of interest, Kouadio *et al.* [187] as well as Ficheux *et al.* [166] showed additive cytotoxicity on Caco-2 and CFU-GM respectively, whereas Wan *et al.* [209] and Bensassi *et al.* [156] observed antagonism on IPEC-J2 and human HCT116 cells respectively, like for NIV+ZEA and DON+NIV+ZEA [209]. Ficheux *et al.* [166] and Bouaziz *et al.* [158] showed the additivity of T2+ZEA on CFU-GM and Vero cells. Wan *et al.* also studied DON+ZEA+FB1, NIV+ZEA+FB1 and DON+NIV+ZEA+FB1 mixtures, and observed the same effects, namely antagonism at the lowest dose (0.5 µM DON and NIV, 10 µM ZEA and 20µM FB1)and synergism at the highest dose (2 µM DON and NIV, and 40 µM ZEA and FB1), as all the other mixtures they studied on IPEC-J2 [209], whereas Kouadio *et al.* showed additivity for DON+ZEA+FB1 on Caco-2 [187].

Concerning the mixtures involving ZEA, FB1 and emerging mycotoxins such as BEA and ENs, a major part presented antagonistic or additive cytotoxic effects. In particular, ZEA and its derivatives α- and β-zearalenol (α-ZOL and β-ZOL) in binary and ternary mixtures were studied by Wang *et al.* [208] and Tatay *et al.* [204]. Wang *et al.* showed mainly an antagonistic effect of ZEA+α-ZOL on HepG2 [208], whereas Tatay *et al.* mostly observed additivity between ZEA and its derivatives on CHO-K1 [204]. Regarding ZEA+FB1, Kouadio *et al.* [187] and Wan *et al.* [209] observed antagonistic effects on Caco-2 and IPEC-J2. Klarić *et al.* showed additivity of FB1+BEA at the lowest concentration (about 0.06 µM BEA and FB1) and synergism at the highest dose (about 6 µM BEA and FB1) on PK15 cells [180]. Concerning emerging mycotoxin mixtures, Ficheux *et al.* studied BEA+ENB and observed additivity on CFU-GM after 14 days [166]. Finally, several authors [189,196] studied binary, ternary, and quaternary EN mixtures (ENA, ENA1, ENB, and ENB1) and in similar concentrations, with the same cell viability assessment and time of exposure (MTT assay, during 24 h). Globally, Lu *et al.* observed synergistic effects at low cytotoxicity levels (IC_25_) and additivity at medium and high inhibitory concentration levels (IC_50_–IC_90_) on CHO-K1 [189,196], whereas Prosperini *et al.* indicated antagonism at low cytotoxicity levels (IC_5_–IC_25_) and additivity at medium and high inhibitory concentration levels (IC_50_–IC_90_) on Caco-2 cells [189,196], highlighting, once again, the influence, among other, of the type of cell used.

Thus, observed effects are not necessarily dose- and time-dependent. For example, the studies of ENA+ENA1 combined effects by Lu *et al.* and Prosperini *et al.* showed opposite conclusions on CHO-K1 and Caco-2 cells respectively, after 24 h exposure and at the same ENA and ENA1 concentrations [189,196]. Moreover, for a same cell model, interspecies and intraspecies sensitivity depends on tested mixtures. For example, concerning intestinal epithelial cells, exposure effect to DON+NIV were antagonist at low doses (0.5–2 µM) for IPEC-J2 (porcine jejunal epithelial cells) and synergistic for IPEC-1 (mix of porcine jejunal and ileal epithelial cells) and human Caco-2 cells. Another observation is for a model cell culture like Caco-2 for example, the number of mycotoxins tested in mixtures could not be predictive of a potential additive or synergistic effect. For example, DON+FX as well as DON+NIV and NIV+FX led to synergistic effect but DON+FX+NIV showed antagonistic effect [155].

Currently, the mycotoxin toxicological combined effects are unpredictable based on their individual effects, despite an increasing number of co-exposure studies.

## 5. Conclusion

Mycotoxins are present in a large range of feed and food, all over the world, in different concentrations, mainly depending on mould genetics and physiology, outdoor and indoor environment and climate changes. Even if certain mycotoxins often occur together (e.g., AFs+OTA, AFs+FUM or DON+ZEA), an infinity of mixtures may be found. Therefore, combined toxicity effects are very hard to predict. In addition to being influenced by the type of mycotoxin mixtures and their concentrations, combined toxicity effects depend on the experimental model design: type of cells exposed, time of exposure, ratio used for each mycotoxin in the mixture, endpoints and tests used, as well as chosen statistical model aspects. In general, most of the mycotoxin mixtures lead to additive or synergistic effects, highlighting a significant threat to human and animal health. Moreover, most studies have been carried out over less than three days, at concentrations above the legal limits. There is therefore a lack of data about chronic exposure at sub-toxic mycotoxin concentrations, closer to real food and feed consumption habits. Through a large panel of mycotoxin contamination studies in food and feed around the world, this review constitutes a strong basis of work, allowing for each continent to have an overview of the multicontaminations and to focus on these ones. Diverse publications already showed important combined effects but more studies about relevant mycotoxin combinations should be carried out and especially should be taken into account by the current regulations which only consider so-far mono-exposure data. Finally, the observed diversity of the possible methodological approaches useable (cell models, studied parameters, time and dose exposure, mathematical tools) raises the question of the need for method standardization at an international level allowing for easier data comparison.

## Figures and Tables

**Table 1:** Some mycotoxins of interest and their fungal source, with primary food and feed hosts and endemic regions. References: [1,2,4,24,25,26,27,28,29,30,31,32].

Mycotoxin	Fungi Source	Product of Primary Concern	Geographical Occurrence
AFs * (B1, B2, G1, G2)	*Aspergillus* (*bombycis*, *flavus*, *nomius*, *ochraceoroseus*, *parasiticus*, *parvisclerotigenus*, *pseudotamarii*, *rambellii*, *toxicarius*)	Cereals and cereal-based products (mainly corn), nuts, nut products and seeds, dried fruits, spices, milk and dairy products, meat, eggs	Temperate, tropical and subtropical regions (Southern Asia and Africa)
OTA *	*Aspergillus* (*alliaceus*, *auricomus*, *carbonarius*, *cretensis*, *flocculosus*, *glaucus*, *lacticoffeatus*, *meleus*, *niger*, *ochraceus*, *pseudoelegans*, *roseoglobulosum*, *sclerotioniger*, *sclerotiorum*, *steynii*, *sulphureus*, *westerdijkiae*); *Penicillium* (*nordicum*, *verrucosum*)	Cereals and cereal-based products (mainly rice and wheat), coffee and cocoa beans; wine, beer, dried fruits, spices, meat	From cool-temperate to tropical regions (Northern and Southern America, Northern and Western Europe, Africa and South Asia)
TCTs * (DON, NIV, T-2, HT-2, DAS)	*Fusarium* (*acuminatum*, *armeniacum*, *culmorum*, *crookwellense*, *equisetii*, *graminearum*, *kyushuense*, *langsethiae*, *poae*, *pseudograminearum*, *sambucinum*, *scirpi*, *sporotrichioides*, *venamtum*)	All cereals and cereal-based products	Northern temperate regions (Europe, America and Asia)
ZEA *	*Fusarium* (*crookwellense*, *culmorum*, *equiseti*, *graminearum*, *incarnatum*, *pseudograminearum*, *semitectum*, *sporotrichioides*, *verticillioides*)	All cereals and cereal-based products, and banana	Northern temperate regions (Europe, America and Asia)
FUM * (B1, B2, B3)	*Fusarium* (*anthophilum*, *dlamini*, *fujikuroi*, *globosum*, *napiforme*, *nygamai*, *oxysporum*, *polyphialidicum*, *proliferatum*, *pseudonygamai*, *thapsinum*, *verticillioides*)	Corn, millet, sorghum, rice and their derivatives	Hot-temperate regions (Europe, Africa)
BEA *	*Fusarium* (*acuminatum*, *armeniacum*, *anthophilum*, *avenaceum*, *beomiforme*, *dlamini*, *equiseti*, *fujikuroi*, *globosum*, *langsethiae*, *longipes*, *nygamai*, *oxysporum*, *poae*, *proliferatum*, *pseudoanthophilum*, *sambucinum*, *semitectum*, *sporotrichioides*, *subglutinans*)	All cereals and cereal-based products	Temperate regions (Europe)
ENs * (A, A1, B, B1)	*Fusarium.*(*acuminatum*, *avenaceum*, *langsethiae*, *lateritium*, *poae*, *proliferatum*, *sambucinum*, *sporotrichioides*, *tricinctum*)	All cereals and cereal-based products	Temperate regions (Europe)
MON *	*Fusarium* (*acuminatum*, *avenaceum*, *culmorum*, *equiseti*, *fujikuroi*, *napiforme*, *nygamai*, *oxysporum*, *proliferatum*, *pseudonygamai*, *sporotrichioides*, *subglutinans*, *thapsinum*, *tricinctum*, *verticillioides*)	All cereals and cereal-based products	Temperate regions (Europe)

* Abbreviations: aflatoxins (AFs); ochratoxin A (OTA); trichothecenes (TCTs); deoxynivalenol (DON); nivalenol (NIV); T-2 toxin (T-2); HT-2 toxin (HT2); diacetoxyscirpenol (DAS); zearalenone (ZEA); fumonisins (FUM); beauvericin (BEA); enniatins (ENs); moniliformin (MON).

**Table 2:** *In vitro* interactions between fusariotoxins on cell viability.

Mycotoxin Couples/Cells	Doses (µM)	Exposure	Toxicological Effect		References
**Interaction between TCT**					
**DON+15-ADON**	DON: 0.25–4	48 h	Synergistic	at low inhibitory concentration levels (IC_10, 20, 30_)	[155]
Human epithelial colorectal adenocarcinoma cells: **Caco-2**	15-ADON: 0.25–4		Additive	at medium inhibit concentration levels (IC_40, 50_)	
**DON+15-ADON**	DON: 0.2–15	24 h	Synergistic	from IC_10_ to IC_80_	[154]
Intestinal porcine epithelial cells (ileum + jejunum): **IPEC-1**	15-ADON: 0.2–15				
**DON+3-ADON**	DON: 0.25–4	48 h	Synergistic	at low and medium inhibitory concentration levels (IC_10, 20, 30, 40_)	[155]
Human epithelial colorectal adenocarcinoma cells: **Caco-2**	3-ADON: 0.42–6.67		Additive	at the 50% growth inhibition level (IC_50_)	
**DON+3-ADON** Intestinal porcine epithelial cells (ileum + jejunum): **IPEC-1**	DON: 0.2–15 3-ADON: 2–150	24 h	Antagonistic	at low inhibitory concentration levels (IC_10_–IC_30_)	[154]
			Additive	at medium inhibitory concentration levels (IC_30_–IC_60_)	
			Synergistic	at high inhibitory concentration levels (IC_60_–IC_80_)	
**15-ADON+3-ADON**	15-ADON: 0.25–4	48 h	Synergistic	at low cytotoxicity levels (IC_10, 20, 30_)	[155]
Human epithelial colorectal adenocarcinoma cells: **Caco-2**	3-ADON: 0.42–6.67		Additive	at medium inhibitory concentration levels (IC_40, 50_)	
**15-ADON+3-ADON**	15-ADON: 0.2–15	24 h	Synergistic	at all cytotoxicity levels (IC_10_–IC_80_)	[154]
Intestinal porcine epithelial cells (ileum + jejunum): **IPEC-1**	3-ADON: 2–150				
**DON+15-ADON+3-ADON** Human epithelial colorectal adenocarcinoma cells: **Caco-2**	DON: 0.25–4	48 h	Synergistic	at low cytotoxicity levels (IC_10, 20, 30_)	[155]
	15-ADON: 0.25–4		Additive	at the 40% growth inhibition level (IC_40_)	
	3-ADON: 0.42–6.67		Antagonistic	from the 50% growth inhibition level (IC_50_)	
**DON+NIV**	DON: 10–100	24 h, 48 h and 72 h	Additive	at 50% growth inhibition level (IC_50_)	[194]
Murine monocyte macrophage cells: **J774A.1**	NIV: 10–100				
**DON+NIV**	DON: 0.5–2	48 h	Antagonistic	at the lowest dose	[209]
Intestinal porcine epithelial cells (jejunum): **IPEC-J2**	NIV: 0.5–2		Synergistic	at the highest dose	
**DON+NIV**	DON: 0.25–4	48 h	Synergistic	at all cytotoxicity levels (from IC_10_ to IC_50_)	[155]
Human epithelial colorectal adenocarcinoma cells: **Caco-2**	NIV: 0.2–3.2				
**DON+NIV**	DON: 0.2–15	24 h	Synergistic	at all cytotoxicity levels (from IC_10_ to IC_80_)	[154]
Intestinal porcine epithelial cells (ileum + jejunum): **IPEC-1**	NIV: 0.2–15				
**DON+FX**	DON: 0.25–4	48 h	Synergistic	at all cytotoxicity levels (from IC_10_ to IC_50_)	[155]
Human epithelial colorectal adenocarcinoma cells: **Caco-2**	FX: 7.5–120				
**DON+FX**	DON: 0.2–15	24 h	Antagonistic	at all inhibitory concentration levels (IC_10_-IC_80_)	[154]
Intestinal porcine epithelial cells (ileum + jejunum): **IPEC-1**	FX: 0.12–9				
**NIV+FX**	NIV: 0.2–3.2	48 h	Synergistic	at low cytotoxicity levels (IC_10, 20_)	[155]
Human epithelial colorectal adenocarcinoma cells: **Caco-2**	FX: 7.5–120		Additive	at medium cytotoxicity levels (IC_30, 40, 50_)	
**NIV+FX**	NIV: 0.2–15	24 h	Additive	at all cytotoxicity levels (IC_10_-IC_80_)	[154]
Intestinal porcine epithelial cells (ileum + jejunum): **IPEC-1**	FX: 0.16–12				
**DON+NIV+FX** Human epithelial colorectal adenocarcinoma cells: **Caco-2**	DON: 0.25–4	48 h	Antagonistic Additive	at low cytotoxicity levels (IC_10, 20_)	[155]
	NIV: 0.2–3.2			at medium cytotoxicity levels (IC_30, 40, 50_)	
	FX: 7.5–120				
**DON+T2**	DON: 0.25–4	24 h, 48 h and 72 h	Antagonistic		[197]
Chinese hamster ovary cells: **CHO-K1**	T2: 0.006–0.1				
**DON+T2**	DON: 0.25–8	24 h, 48 h and 72 h	Antagonistic		[198]
Monkey kidney epithelial cells: **Vero**	T2: 0.001–0.05				
**DON+T2**	DON: 0.04–0.1	14 days	Additive		[166]
Hematopoietic progenitors: **CFU-GM**	T2: 0.0005–0.0016				
**Interaction between TCT and other fusariotoxins**					
**DON+BEA**	DON: 0.25–4	24 h, 48 h and 72 h	Antagonistic		[197]
Chinese hamster ovary cells: **CHO-K1**	BEA: 0.78–12.5				
**DON+BEA**	DON: 0.25–8	24 h, 48 h and 72 h	Antagonistic		[198]
Monkey kidney epithelial cells: **Vero**	BEA: 0.78–25				
**DON+BEA**	DON: 0.04–0.1	14 days	Synergistic		[166]
Hematopoietic progenitors: **CFU-GM**	BEA: 0.064–3.2				
**T2+BEA**	T2: 0.006–0.1	24 h, 48 h and 72 h	Synergistic		[197]
Chinese hamster ovary cells: **CHO-K1**	BEA: 0.78–12.5				
**T2+BEA**	T2: 0.001–0.05	24 h, 48 h and 72 h	Antagonistic		[198]
Monkey kidney epithelial cells: **Vero**	BEA: 0.78–25				
**DON+FB1**	DON: 4–20	72 h	Additive		[187]
Human epithelial colorectal adenocarcinoma cells: **Caco-2**	FB1: 10				
**DON+FB1**	DON: 0.04–0.1	14 days	Antagonistic		[166]
Hematopoietic progenitors: **CFU-GM**	FB1: 0.5–2				
**DON+FB1**	DON: 0.5–2	48 h	Antagonistic	at the lowest dose	[209]
Intestinal porcine epithelial cells (jejunum): **IPEC-J2**	FB1: 20–40		Synergistic	at the highest dose	
**NIV+FB1**	NIV: 0.5–2	48 h	Antagonistic	at the lowest dose	[209]
Intestinal porcine epithelial cells (jejunum): **IPEC-J2**	FB1: 20–40		Synergistic	at the highest dose	
**DON+ZEA**	DON: 10–20	72 h	Additive		[187]
Human epithelial colorectal adenocarcinoma cells: **Caco-2**	ZEA: 10–20				
**DON+ZEA**	DON: 0.04–0.1	14 days	Additive		[166]
Hematopoietic progenitors: **CFU-GM**	ZEA: 0.2–10				
**DON+ZEA**	DON: 0.5–2	48 h	Antagonistic	at the lowest dose	[209]
Intestinal porcine epithelial cells (jejunum): **IPEC-J2**	ZEA: 10–40		Synergistic	at the highest dose	
**DON+ZEA**	DON: 100	24 h	Antagonistic		[156]
Human colon carcinoma cells: **HCT116**	ZEA: 40				
**NIV+ZEA**	NIV: 0.5–2	48 h	Antagonistic	at the lowest dose	[209]
Intestinal porcine epithelial cells (jejunum): **IPEC-J2**	ZEA: 10–40		Synergistic	at the highest dose	
**T2+ZEA**	T2: 0.0005–0.0016	14 days	Additive		[166]
Hematopoietic progenitors: **CFU-GM**	ZEA: 0.2–10				
**T2+ZEA**	T2: 0.025–0.1	24 h	Additive		[158]
Monkey kidney epithelial cells: **Vero**	ZEA: 0.025–0.1				
**DON+T2+BEA** Chinese hamster ovary cells: **CHO-K1**	DON: 0.25–4	24 h, 48 h, and 72 h	Synergistic		[197]
	T2: 0.006–0.1				
	BEA: 0.78–12.5				
**DON+T2+BEA** Monkey kidney epithelial cells: **Vero**	DON: 0.25–8	24 h, 48 h, and 72 h	Antagonistic		[198]
	T2: 0.001–0.05				
	BEA: 0.78–25				
**DON+NIV+ZEA** Intestinal porcine epithelial cells (jejunum): **IPEC-J2**	DON: 0.5–2	48 h	Antagonistic	at the lowest dose	[209]
	NIV: 0.5–2				
	ZEA: 10–40		Synergistic	at the highest dose	
**DON+NIV+FB1**	DON: 0.5–2	48 h	Antagonistic	at the lowest dose	[209]
	NIV: 0.5–2				
Intestinal porcine epithelial cells (jejunum): **IPEC-J2**	FB1: 20–40		Synergistic	at the highest dose	
**DON+ZEA+FB1** Human epithelial colorectal adenocarcinoma cells: **Caco-2**	DON: 10–20 ZEA: 10–20 FB1: 10	72 h	Additive		[187]
**DON+ZEA+FB1** Intestinal porcine epithelial cells (jejunum): **IPEC-J2**	DON: 0.5–2Z EA: 10–40 FB1: 20–40	48 h	Antagonistic Synergistic	at the lowest dose at the highest dose	[209]
**NIV+ZEA+FB1** Intestinal porcine epithelial cells (jejunum): **IPEC-J2**	NIV: 0.5–2 ZEA: 10–40 FB1: 20–40	48 h	Antagonistic Synergistic	at the lowest dose at the highest dose	[209]
**DON+NIV+ZEA+FB1** Intestinal porcine epithelial cells (jejunum): **IPEC-J2**	DON: 0.5–2 NIV: 0.5–2 ZEA: 10–40 FB1: 20–40	48 h	Antagonistic Synergistic	at the lowest dose at the highest dose	[209]
**Interaction between other fusariotoxins**					
**ZEA+α-ZOL** Human hepatocellular carcinoma cells: **HepG2**	ZEA: 0.5–50 α-ZOL: 1–100	24 h and 72 h	Antagonistic	at all cytotoxicity levels (from IC_10_ to IC_90_)	[208]
		48 h	Antagonistic	at IC_10, 20, 30, 40_	
			Additive	at IC_50, 60, 70_	
			Synergistic	at IC_80, 90_	
**ZEA+α-ZOL** Chinese hamster ovary cells: **CHO-K1**	ZEA: 12.5–50 α-ZOL: 6.25–25	24 h	Synergistic	at low cytotoxicity level (IC_25_)	[204]
			Additive	at medium and high cytotoxicity levels (from IC_50_ to IC_90_)	
		48 h and 72 h	Additive	at all cytotoxicity levels (from IC_25_ to IC_90_)	
**ZEA+β-ZOL** Chinese hamster ovary cells: **CHO-K1**	ZEA: 12.5–50 β-ZOL: 6.25–25	24 h, 48 h and 72 h	Additive	at all cytotoxicity levels (from IC_25_ to IC_90_)	[204]
**α-ZOL+β-ZOL** Chinese hamster ovary cells: **CHO-K1**	α-ZOL: 6.25–25	24 h	Additive	at all cytotoxicity levels (IC_25, 50, 75, 90_)	[204]
		48 h	Antagonistic	at low and medium cytotoxicity levels (IC_25, 50_)	
	β-ZOL: 6.25–25		Additive	at high cytotoxicity levels (IC_75, 90_)	
		72 h	Additive	at low and high cytotoxicity levels (IC_25, 75, 90_)	
			Antagonistic	at medium cytotoxicity level (IC_50_)	
**ZEA+α-ZOL+β-ZOL**	ZEA: 12.5–5	24 h and 48 h	Antagonistic	at low and medium cytotoxicity levels (IC_25, 50_)	[204]
			Synergistic	at high cytotoxicity levels (IC_75, 90_)	
Chinese hamster ovary cells: **CHO-K1**	α-ZOL: 6.25–25 β-ZOL: 6.25–25	72 h	Antagonistic	at low cytotoxicity level (IC_25_)	
			Synergistic	at medium and high cytotoxicity levels (IC_50, 75, 90_)	
**ZEA+FB1** Human epithelial colorectal adenocarcinoma cells: **Caco-2**	ZEA: 5–20 FB1: 10	72 h	Antagonistic		[187]
**ZEA+FB1** Intestinal porcine epithelial cells (jejunum): **IPEC-J2**	ZEA: 10–40 FB1: 20–40	48 h	Antagonistic	at the lowest dose	[209]
			Synergistic	at the highest dose	
**ZEA+FB1** Human epithelial colorectal adenocarcinomia cells: **Caco-2**	ZEA: 10 FB1: 10	72 h	Antagonistic		[186]
**BEA+FB1** Porcine renal proximal tubular epithelial cells: **PK15**	BEA: 0.064–6.4 µM FB1: 0.069–6.9 µM	24 h	Additive	at low doses	[179]
			Antagonistic	at the highest dose	
**BEA+ENB** Hematopoietic progenitors: **CFU-GM**	BEA: 0.064–3.2 ENB: 2–6	14 days	Additive		[166]
**ENA+ENA_1_** Chinese hamster ovary cells: **CHO-K1**	ENA: 0.365–5 ENA_1_: 0.625–5	24 h	Synergistic	at low cytotoxicity levels (IC_25_)	[189]
			Additive	at medium and high cytotoxicity levels (IC_50, 75, 90_)	
**ENA+ENA_1_** Human epithelial colorectal adenocarcinomia cells: **Caco-2**	ENA: 0.365–5 ENA_1_: 0.625–5	24 h	Antagonistic	at the lowest fraction affected (IC_5_)	[196]
			Additive	at other fractions affected (IC_25_, _50_, _75_, _90_)	
**ENA+ENB** Chinese hamster ovary cells: **CHO-K1**	ENA: 0.365–5 ENB: 0.625–5	24 h	Synergistic	at low and medium cytotoxicity levels (IC_25, 50_)	[189]
			Additive	at high cytotoxicity levels (IC_75, 90_)	
**ENA+ENB** Human epithelial colorectal adenocarcinomia cells: **Caco-2**	ENA: 0.365–5 ENB: 0.625–5	24 h	Antagonistic	at the lowest fraction affected (IC_5_)	[196]
			Additive	at other fractions affected (IC_25_, _50_, _75_, _90_)	
**ENA+ENB_1_** Chinese hamster ovary cells: **CHO-K1**	ENA: 0.365–5 ENB_1_: 0.625–5	24 h	Additive	at all inhibitory concentration levels (IC_25, 50, 75, 90_)	[189]
**ENA+ENB_1_** Human epithelial colorectal adenocarcinomia cells: **Caco-2**	ENA: 0.365–5 ENB_1_: 0.625–5	24 h	Antagonistic	at the lowest fraction affected (IC_5_)	[196]
			Additive	at other fractions affected (IC_25_, _50_, _75_, _90_)	
**ENA_1_+ENB**	EN A_1_: 0.365–5	24 h	Additive	at all inhibitory concentration levels (IC_25, 50, 75, 90_)	[189]
Chinese hamster ovary cells: **CHO-K1**	ENB: 0.625–5				
**ENA_1_+ENB** Human epithelial colorectal adenocarcinoma cells: **Caco-2**	EN A_1_: 0.365–5 ENB: 0.625–5	24 h	Antagonistic	at the lowest fraction affected (IC_5_)	[196]
			Additive	at medium fractions affected (IC_25_, _50_, _75_)	
			Synergistic	at the highest fraction affected (IC_90_)	
**ENA_1_+ ENB_1_**	EN A_1_: 0.365–5	24 h	Synergistic	at low, medium and high cytotoxicity levels (IC_25, 50, 75_)	[189]
Chinese hamster ovary cells: **CHO-K1**	ENB_1_: 0.625–5		Additive	at very high cytotoxicity levels (IC _90_)	
**ENA_1_+ ENB_1_** Human epithelial colorectal adenocarcinoma cells: **Caco-2**	EN A_1_: 0.365–5 ENB_1_: 0.625–5	24 h	Additive Synergistic	at the lowest fraction affected (IC_5_)	[196]
				at medium fractions affected (IC_25_, _50_)	
				at the two highest fractions affected (IC_75_, _90_)	
**ENB+ENB_1_**	ENB: 0.365–5	24 h	Additive	at all inhibitory concentration levels (IC_25, 50, 75, 90_)	[189]
Chinese hamster ovary cells: **CHO-K1**	ENB_1_: 0.625–5				
**ENB+ENB_1_** Human epithelial colorectal adenocarcinoma cells: **Caco-2**	ENB: 0.365–5ENB_1_: 0.625–5	24 h	Antagonistic	at the two lowest fractions affected (IC_5, 25_)	[196]
			Additive	at other fractions affected (IC_50_, _75_, _90_)	
**ENA+ENA_1_+ENB** Chinese hamster ovary cells: **CHO-K1**	ENA: 0.3125–2.5	24 h	Synergistic Additive	at low and medium cytotoxicity levels (IC_25, 50_) at high cytotoxicity levels (IC_75, 90_)	[189]
	ENA_1_: 0.3125–2.5				
	ENB: 0.3125–2.5				
E**NA+ENA_1_+ENB** Human epithelial colorectal adenocarcinoma cells: **Caco-2**	ENA: 1.25–5	24 h	Antagonistic	at the lowest fraction affected (IC_5_)	[196]
	ENA_1_: 1.25–5		Additive	at medium fractions affected (IC_25_, _50_)	
	ENB: 1.25–5		Synergistic	at the two highest fractions affected (IC_75_, _90_)	
**ENA+ENA_1_+ENB_1_** Chinese hamster ovary cells: **CHO-K1**	ENA: 0.3125–2.5	24 h	Synergistic	at low and medium cytotoxicity levels (IC_25, 50_)	[189]
	ENA_1_: 0.3125–2.5		Additive	at high cytotoxicity level (IC_75_)	
	ENB_1_: 0.3125–2.5		Antagonistic	at very high cytotoxicity level (IC_90_)	
**ENA+ENA_1_+ENB_1_** Human epithelial colorectal adenocarcinoma cells: **Caco-2**	ENA: 1.25–5	24 h	Antagonistic Additive	at the lowest fraction affected (IC_5_) at other fractions affected (IC_25_, _50_, _75_, _90_)	[196]
	ENA_1_: 1.25–5				
	ENB_1_: 1.25–5				
**ENA+ENB+ENB_1_** Chinese hamster ovary cells: **CHO-K1**	ENA: 0.3125–2.5	24 h	Synergistic Additive	at low and medium cytotoxicity levels (IC_25, 50_) at high cytotoxicity levels (IC_75, 90_)	[189]
	ENB: 0.3125–2.5				
	ENB_1_: 0.3125–2.5				
**ENA+ENB+ENB_1_** Human epithelial colorectal adenocarcinoma cells: **Caco-2**	ENA: 1.25–5	24 h	Antagonistic Additive	at the lowest fraction affected (IC_5_) at other fractions affected (IC_25_, _50_, _75_, _90_)	[196]
	ENB: 1.25–5				
	ENB_1_: 1.25–5				
**ENA_1_+ENB+ENB_1_** Chinese hamster ovary cells: **CHO-K1**	ENA_1_: 0.3125–2.5	24 h	Synergistic	at low cytotoxicity level (IC_25_)	[189]
	ENB: 0.3125–2.5		Additive	at medium and high cytotoxicity levels (IC_50,__75_)	
	ENB_1_: 0.3125–2.5		Antagonistic	at very high cytotoxicity level (IC_90_)	
**EN A_1_+ENB+ENB_1_** Human epithelial colorectal adenocarcinoma cells: **Caco-2**	ENA_1_: 1.25–5	24 h	Antagonistic Additive	at the lowest fraction affected (IC_5_) at other fractions affected (IC_25_, _50_, _75_, _90_)	[196]
	ENB: 1.25–5				
	ENB_1_: 1.25–5				
**ENA+ENA_1_+ENB+ENB_1_** Human epithelial colorectal adenocarcinoma cells: **Caco-2**	ENA: 1.25–5	24 h	Antagonistic Additive	at the lowest fraction affected (IC_5_) at other fractions affected (IC_25_, _50_, _75_, _90_)	[196]
	ENA_1_: 1.25–5				
	ENB: 1.25–5				
	ENB_1_: 1.25–5				

Abbreviations: deoxynivalenol (DON); 3-acetyldeoxynivalenol (3-ADON); 15-acetyldeoxynivalenol (15-ADON); nivalenol (NIV); fusarenone-X (FUS-X); T-2 toxin (T-2); beauvericin (BEA); fumonisin B1 (FB1); zearalenone (ZEA); α-zearalenol (α-ZOL); β-zearalenol (β-ZOL); enniatins A, A1, B, B1 (ENA, ENA1,ENB, ENB1)

## Abbreviations

The following abbreviations are used in this manuscript:

3-ADON	3-acetyldeoxynivalenol
15-ADON	15-acetyldeoxynivalenol
α-ZOL	α-zearalenol
β-ZOL	β-zearalenol
AFs	aflatoxins
BEA	beauvericine
DAS	diacetoxyscirpenol
DON	deoxynivalenol
EC	European Commission
EFSA	European Food Safety Authority
ENA, ENA1,ENB, ENB1	enniatins A, A1, B, B1
ENs	enniatins
FAO	Food and Agriculture Organization
FDA	Food and Drug Administration
FB1, FB2, FB3	fumonisin B1, B2, B3
FUM	fumonisins
FUS-X	fusarenone-X
IC	inhibitory concentration
HT-2	HT-2 toxin
MON	moniliformin
NIV	nivalenol
OTA	ochratoxin A
TCTs	trichothecenes
T-2	T-2 toxin
ZEA	zearalenone
WHO	World Health Organization
